# U-shaped association between triglyceride and risk of incident diabetes in normoglycemic males with NAFLD: A population-base cohort study

**DOI:** 10.7150/ijms.83371

**Published:** 2023-09-04

**Authors:** Xuemei Xie, Jixiang Liao, Chenghu Huang, Xiaowei Li, Qiuli Cao, Lijuan Kong, Takuro Okamura, Yoshitaka Hashimoto, Akihiro Obora, Takao Kojima, Michiaki Fukui, Masahide Hamaguchi, Zuojie Luo, Yingfen Qin, Xinghuan Liang, Xiuping Xuan

**Affiliations:** 1 Department of Endocrinology, The First Affiliated Hospital of Guangxi Medical University, Nanning, Guangxi, China.; 2 Transplant Medical Center, The Second Affiliated Hospital of Guangxi Medical University, Nanning, Guangxi 530007, China.; 3 Guangxi Key Laboratory of Organ Donation and Transplantation, Nanning, Guangxi 530007, China.; 4 Guangxi Transplantation Medicine Research Center of Engineering Technology, Nanning, Guangxi 530007, China.; 5 Department of Endocrinology, Bishan Hospital of Chongqing, Bishan, Chongqing, China.; 6 Department of Endocrinology and Metabolism, Kyoto Prefectural University of Medicine, 465 Kajii-cho, Kawaramachi-Hirokoji, Kamigyo-ku, Kyoto, Japan.; 7 Department of Gastroenterology, Asahi University Hospital, 3-23, Hashimoto-cho, Gifu, Japan.

**Keywords:** triglyceride, Incident type 2 diabetes mellitus, U-shaped association, NAFLD

## Abstract

**Background:** Serum triglyceride (TG) was an important biomarker for nonalcoholic fatty liver disease (NAFLD), and the association between TG and incident type 2 diabetes mellitus is still under debate with some studies suggesting that elevated TG increase the risk of incident T2DM while others indicative of a negative relationship. These controversial findings may be partially due to the inclusion of the participants with NAFLD. The association between TG and incident type 2 diabetes mellitus in people with NAFLD remained unclear. Therefore, this study aimed to characterize the relationship between the baseline TG levels and incident type 2 diabetes mellitus in a male Japanese cohort with NAFLD.

**Methods:** A total of 1221 males with NAFLD were enrolled from the Nagala (NAFLD in the Gifu Area Longitudinal analysis) study conducted from 2004 to 2015. Cox proportional hazards models were performed to examine the relationship between baseline TG concentration and incident type 2 diabetes mellitus. A two-piecewise linear regression model was explored to evaluate the threshold effect of the baseline TG levels on type 2 diabetes mellitus incidence by using a smoothing function.

**Results:** During a median follow-up of 6.05 years, 39 males with NAFLD at baseline developed type 2 diabetes mellitus. The risk of incident type 2 diabetes mellitus was significantly associated with baseline TG concentration in males with NAFLD after fully adjustment for confounders, with per 10 mg/dl elevation in TG levels increasing the risk of incident diabetes by 8.5% (HR=1.085, CI=1.039-1.132; P<0.001). However, no typical dose-dependent positive association between type 2 diabetes mellitus incidence and the TG levels was observed across the TG tertiles. Interestingly, a U-shaped association between TG concentration and risk of incident type 2 diabetes mellitus was revealed by the two-piecewise linear regression analysis. Baseline TG concentration lower than the threshold values (TG <53mg/dl) were negatively associated with risk of incident type 2 diabetes mellitus. With each 10mg/dl increase in baseline TG levels, the risk of incident type 2 diabetes mellitus decreased by nearly 59% (HR=0.413, 95% CI=0.220-0.778). In contrast, when TG levels were higher than the threshold values (TG>53mg/dl), the risk of incident diabetes increased 9.1% with every 10mg TG elevation (HR=1.091, 95% CI=1.046-1.137).

**Conclusions:** A U-shaped relationship was observed between baseline TG levels and incident type 2 diabetes mellitus in a male normoglycemic Japanese population with NAFLD, although extrapolation of the finding to other populations should be made with caution.

## Introduction

Nonalcoholic fatty liver disease (NAFLD) is currently considered as the most common chronic liver disease 1-3, affecting 25% of the global adult population 2-5. NAFLD is characterized by excess triglyceride (TG) accumulation in the liver 6 and may progress from steatosis, often defined as nonalcoholic fatty liver (NAFL), to nonalcoholic steatohepatitis (NASH) 3, 6, 7. With ongoing liver damage, some patients will develop various liver-related complications, including cirrhosis and hepatocellular carcinoma 3, 6, 8.

NAFLD is associated not only with increased liver-related morbidity and mortality, but also with an increased risk of developing other extra-hepatic diseases, such as type 2 diabetes 1, 2, 6. There were evidences showing that NAFLD may precede and/or promote the development of type 2 diabetes mellitus 1, 9. Thus, knowledge about the underlying risk factors for developing glucose dysregulation in individuals with NAFLD may enlighten viable strategies to prevent type 2 diabetes mellitus incidences.

Serum biomarkers, such as TG, is a risk factor for NAFLD and commonly used to assess the severity of NAFLD 3, 7, 10-12. Kashyap SR et al found that TG levels strongly associated with advanced stages of NAFLD and NASH, and TG >150 mg/dl increased the risk of NASH by 3.4-fold in an obesity surgery cohort 11. Consistently, Cao W et al. investigated that TG was a risk factor for progression from NAFL to NASH in a Chinese population 13. There were evidences that elevated TG levels increase the risk of incident type 2 diabetes mellitus 14-20. Interestingly, some studies also indicated that TG levels were negatively correlated with incident diabetes 21-23. The associations between TG and incidence of type 2 diabetes mellitus were still controversial. Previous studies describing the relation between TG and incident diabetes, did not consider the presence of NAFLD; thus, the aforementioned studies may have inevitably included participants with or without NAFLD, a risk factor for diabetes, which may confound the interpretation. The association between TG and incident diabetes in people with NAFLD remained unclear.

This study, therefore, aimed to investigate and characterize the associations between the baseline TG levels and incident diabetes in a normoglycemic Japanese population with NAFLD.

## Methods

### Design and participants

All these data were derived from the NAGALA (NAFLD in the Gifu Area, Longitudinal Analysis) database, which has been described in detail previously 24. In brief, the NAGALA was a population-based longitudinal cohort study of a medical examination program at Murakami Memorial Hospital (Gifu, Japan) from May 1st, 1994 to Dec 31st, 2016, aiming to detect chronic diseases and their risk factors, and contribute to promote public health 24. Most participants in the program received one to two medical exams per year 24. Data from individuals participating in the medical examination program at Murakami Memorial Hospital from 2004 to 2015 were extracted. Participants with known other liver diseases other than NAFLD (viral, autoimmune, genetic, etc), any medication usage, diabetes, prediabetes, as well as missing data of covariates at baseline were excluded. Among them, individuals with 5.7% ≤HbA1C< 6.5% or 5.6 mmol/l ≤FPG <7.0 mmol/l were defined as prediabetes 25. Eventually, 6152 males were included. And subsequently, 1341 males with fatty liver were further extracted. Moreover, to eliminate participants with alcoholic fatty liver disease, 120 males with a daily alcohol consumption ≥ 30 g were excluded 8. Finally, 1221 males with NAFLD at baseline were enrolled in this study. This study was approved by the ethics committee of Murakami Memorial Hospital.

### Data collection and measurement

Data collection and measurement has been described in the previous study 24. Lifestyle factors (including smoking, alcohol habits and physical activity), family history of diabetes, and the medical history were acquired by a standardized self-administered questionnaire. The mean weekly ethanol intake was evaluated by the volume and the type of alcohol consumption per week in the previous month. For smoking status, the participants were categorized into three groups: never, ex or current. Non-smokers were defined as individuals who never smoked, ex-smokers as participants who had smoked cigarettes in the past but quitted until baseline, and current-smokers were referred to participants who smoked at baseline visit. Recreational and sports activities of participants were determined by the questionnaire 26. Regular exercisers were referred to participants who regularly played any type of sports more than once a week 26.

### Definition of NAFLD

Fatty liver was assayed by the findings of abdominal ultrasonography and diagnosed by gastroenterologists who were blind to the participants' personal data. The four known criteria for diagnosing fatty liver were used 24, 26: (1) hepatorenal echo contrast; (2) liver brightness; (3) deep attenuation; and (4) vascular blurring. Fatty liver without significant alcohol abuse (less than 210 g per week in males) was defined as NAFLD 8, 26.

### Exposure

The exposure in this study was fasting TG concentration of participants at baseline.

### Primary outcomes

Incident type 2 diabetes was defined as FPG ≥7mmol/l or HbA1c≥6.5% according to the diagnostic criteria of ADA or self-reported 25.

### Statistical analyses

**Figure 1:**
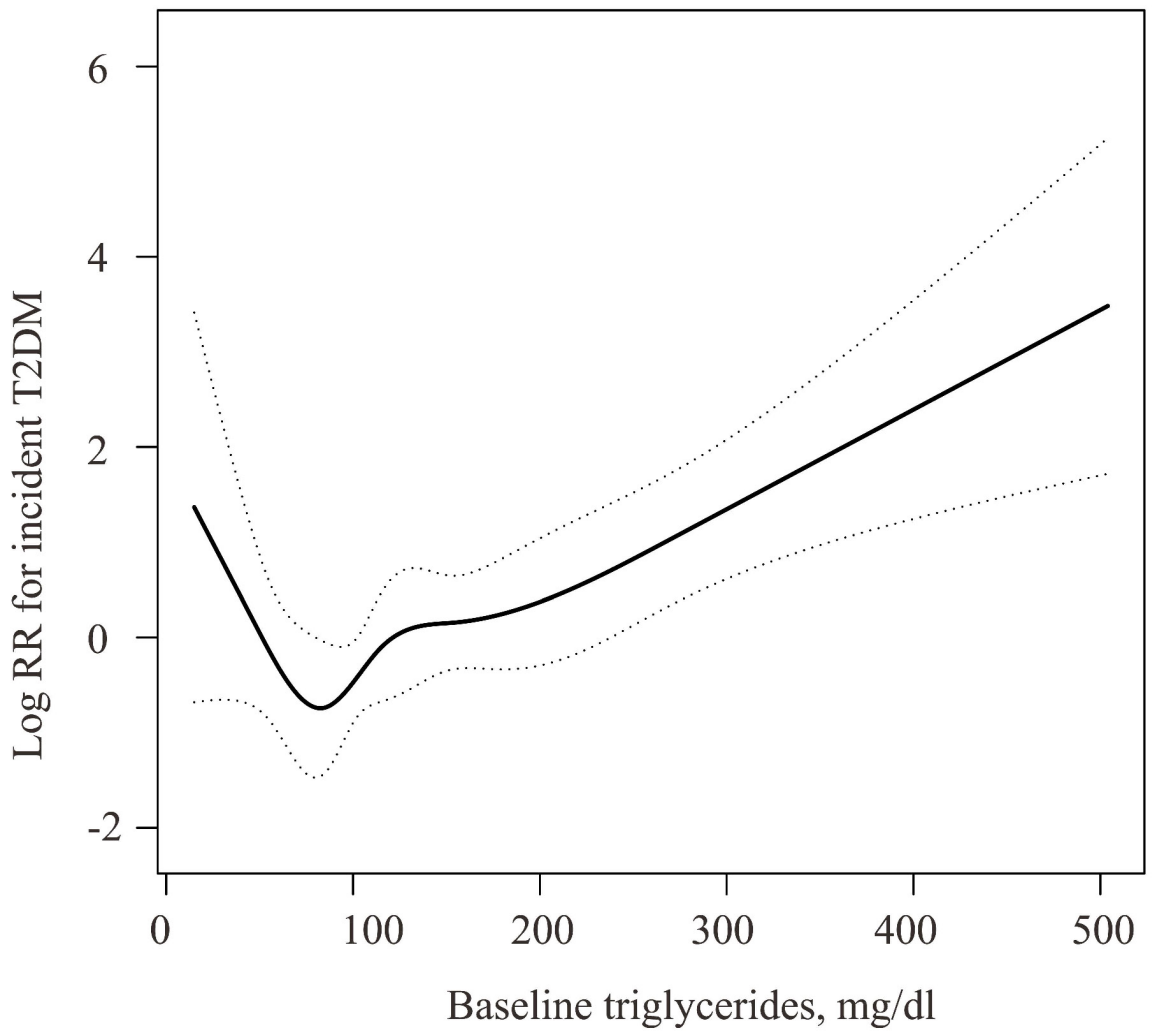
U-shape relationship of the TG and incident diabetes in normoglycemic men with NAFLD. The solid black line was the smooth curve fit between the TG and incident diabetes. The dotted curves represented the 95% CI of the fit. The association was adjusted for Age, BMI (body mass index), Waist circumference, Body Weight, ethanol consumption (g/week), ALT, AST, regular exercise, GGT, HDL-cholesterol, Total Cholesterol, HbA1c, smoking status, fasting plasma glucose, systolic blood pressure, diastolic blood pressure, and father or mother with diabetes.

Baseline characteristics of participants were classified according to the TG tertiles. Continuous variables are presented as mean (S.D.) or as median (Q1-Q3), while categorical data are presented as number (percentage). Data normality were explored by Kolmogorov-Smirnov tests. For continuous normally distributed variables, statistical differences among groups were evaluated using one-way analysis of variance. And for skewed distributed data, Kruskal Wallis test was used (Table 1). The chi-square test was used to compare categorical variables (Table 1). Univariable logistic regression analysis was performed to evaluate the potential effect of age, BMI, waist circumference, body weight, ethanol consumption, ALT, AST, GGT, HDL-cholesterol, total cholesterol, TG, HbA1c, FPG, systolic blood pressure, diastolic blood pressure, and family history of diabetes on incident type 2 diabetes mellitus, respectively (Table 2). Cox proportional hazards models were used to evaluate the relationship between baseline TG concentration and incident type 2 diabetes mellitus with or without adjustment for the following potential covariates: age, BMI, waist circumference, body weight, ethanol consumption, ALT, AST, regular exercise, GGT, HDL-cholesterol, total cholesterol, HbA1c, smoking status, FPG, systolic blood pressure, diastolic blood pressure, and father or mother with diabetes (Table 3). We then applied a two-piecewise linear regression model to examine the threshold effect of the log TG on incident diabetes using a smoothing function (Figure 1, Table 4). A log likelihood ratio test was conducted to compare the online linear regression model with a two-piecewise linear model. Results were considered statistically significant with P value < 0.05 (two-tailed). All statistical analyses were performed by the statistical packages R (The R Foundation; http://www.r-project.org; version 3.6.1) and EmpowerStats^27^.

## Results

### Baseline characteristics

A total of 1221 males with NAFLD at baseline were enrolled in the present study. The baseline characteristics of the study cohort were showed in Table 1 according to the TG tertiles. The body weight, waist circumference, BMI (body mass index), ethanol consumption, ALT, AST levels, GGT concentration, HDL-cholesterol, TG levels, systolic blood pressure, diastolic blood pressure, and the number (percentage) of family history of diabetes were the highest in participants within the third tertile of TG (all P <0.05, Table 1). However, the baseline age, HbA1c, FPG, and the number (percentage) of regular exercise and family history of diabetes did not differ significantly among the three groups (P > 0.05, Table 1). During the median follow-up period of 6.05 years, 39 males with NAFLD at baseline developed type 2 diabetes mellitus.

### Univariate analysis of the relationship between baseline characteristics and incident type 2 diabetes mellitus

The univariate regression analysis of the association between baseline characteristics and incident type 2 diabetes mellitus suggested that age, waist circumference, BMI, TG concentration, HbA1c levels, and father or mother with diabetes were all unadjusted risk factors for incident diabetes (P<0.05, Table 2). However, body weight, ethanol consumption, HDL-cholesterol, total cholesterol, GGT, FPG, systolic blood pressure, and diastolic blood pressure were not significantly associated with risk of incident diabetes (P > 0.05, Table 2).

### Multivariate analysis of the association between baseline TG concentration and incident type 2 diabetes mellitus

Multivariate regression analysis was performed to assess the independent effects of the baseline TG levels on incident type 2 diabetes mellitus (Table 3). The crude model indicated that per 10 mg/dl elevation in TG levels increased the risk of incident diabetes by 6.6% (hazard ratio [HR] = 1.066, 95% confidence interval [CI] = 1.032 - 1.102; P < 0.001, Table 3). After adjusting for age, BMI, waist circumference and body weight, the association remained significant (HR = 1.068, CI = 1.033 - 1.104; P < 0.001, Adjusted model I, Table 3). Further adjustment for ethanol consumption, ALT, AST, regular exercise, GGT, HDL-cholesterol, total cholesterol, HbA1c, smoking, FPG, systolic blood pressure, diastolic blood pressure, and family history of diabetes did not alter the significant relationship (HR = 1.085, CI = 1.039 - 1.132; P < 0.001, Adjusted model II, Table 3).

The participants were then divided into tertiles according to the baseline TG levels. Compared to the lowest TG Tertlie, the risk of incident type 2 diabetes mellitus did not increase significantly in Tertile 2 (Crude model: HR = 1.374, CI = 0.523 - 3.611; Adjusted Model I: HR = 1.23, CI = 0.46 - 3.25; and Adjusted Model II: HR = 1.418, CI = 0.489 - 4.109, Table 3). However, the risk of incident diabetes increased significantly in Tertile 3 (Crude model: HR = 2.867, CI = 1.224 - 6.713; Adjusted Model I: HR = 2.60, CI = 1.10 - 6.16; and Adjusted Model II: HR = 2.871, CI = 1.001 - 8.234, Table 3). The increased risk of baseline TG elevation on incident diabetes did not show any typical dose-dependent association, indicative of the existence of a nonlinear relationship.

### Two-piecewise linear regression model analysis using a smoothing function

As the above multivariable regression analysis suggested a nonlinear association between the baseline TG levels and risk of incident type 2 diabetes mellitus, a two-piecewise linear regression model was performed to further explore their association using a smoothing function. Interestingly, adjusted smoothed plots identified a U-shaped association between baseline TG levels and incident type 2 diabetes mellitus (Figure 1). According to the two-piecewise linear regression model, the baseline TG magnitude was significantly negatively related to the log relative risk (log [RR]) of incident type 2 diabetes mellitus with TG concentration < 53mg/dl after adjusting for confounders (Figure 1, Table 4). With each 10mg/dl increase in baseline TG levels, the risk of incident type 2 diabetes mellitus decreased by nearly 59% (HR = 0.413, 95% CI = 0.220 - 0.778, Table 4) after adjusting for age, BMI, waist circumference, body weight, ethanol consumption, ALT, AST, GGT, exercise, HDL-cholesterol, total cholesterol, HbA1c, smoking, FPG, systolic blood pressure, diastolic blood pressure, and father or mother with diabetes. With the baseline TG elevating up to the turning point (TG = 53 mg/dl), the risk of type 2 diabetes mellitus decreased to the lowest level (Figure 1, Table 4). However, the baseline TG levels were significantly positively correlated to the risk of incident type 2 diabetes mellitus when TG levels were higher than 53 mg/dl, (HR = 1.091, 95% CI = 1.046 - 1.137, Table 4).

## Discussion

To our best knowledge, the present study first demonstrated a U-shaped association between baseline TG concentration and risk of incident type 2 diabetes mellitus among normoglycemic males with NAFLD at baseline. A turning point of TG at 53mg/dl using the threshold effect analysis after adjustment for potential confounders was revealed (Figure 1, Table 4).

Previous studies have evaluated the association between concentration of TG and incident type 2 diabetes mellitus in nondiabetic persons 14, 16-19, 21-23. Most of the studies suggested the TG was positively linked with incident diabetes 14, 16-19. For example, Ye-Li WANG et al. 14 and Nishikawa T et al. 19 showed that TG levels were positively associated with risk of type 2 diabetes mellitus in participants without diabetes at baseline. Similarly, Jieru Peng et al. also reported that TG was positively related to risk of incident type 2 diabetes mellitus in middle-aged and elderly adults 16. Consistently, in our study, baseline TG at the level of >53 mg/dl was positively associated with the risk of incident diabetes in males without NAFLD (Figure 1, Table 4).

Interestingly, some previous studies also indicated of the protective effect of raised TG levels on type 2 diabetes mellitus 21-23. For instance, TG was negatively correlated with incident diabetes in Mendelian randomization analysis 21. Consistently, Palmer CN et al. reported that severely obese participants carrying the PNPLA3 148M allele had lower serum triglyceride concentration but were more insulin resistant and susceptible to type 2 diabetes mellitus 22. Moreover, Mc Donald Posso AJ found that TG ≥ 150 mg/dL were positively associated with diabetes in both genders, while the relationship was inverse with TG < 150 mg/dL 15, suggesting a nonlinear association. However, whether a U-shaped association between TG and incident type 2 diabetes mellitus was existed had not been explored. These results were in line with the current study that, lower baseline TG levels (TG < 53mg/dl) substantially changed the relationship between the baseline TG concentration and risk of incident type 2 diabetes mellitus (Figure 1, Table 4). In individuals with TG lower than the threshold, the risk of incident type 2 diabetes mellitus in males with NAFLD decreased by nearly 59%, with each 10mg/dl increment in the baseline TG levels after fully adjusting for confounders (Figure 1, Table 4).

In the previous studies on the associations between TG levels and incident diabetes, there was no clinical data on fatty liver, which means that the aforementioned studies may have inevitably included participants with or without NAFLD 14-19, 21-23. With the U-shaped association between the TG and incident diabetes in NAFLD in this study, we speculated that, fatty liver, a risk factor for diabetes, may have confound the results and lead to inconsistent interpretation on the relationships between TG and diabetes in the previous reports. Indeed, we have also explored the correlation between baseline TG and incident type 2 diabetes mellitus in normoglycemic males without NAFLD, and no U-shaped association was found (unpublished data).

The underlying mechanisms of the U-shape associations between TG and type 2 diabetes mellitus were not fully known. The positive association between TG and the risk of incident type 2 diabetes mellitus was possibly due to adverse effect of hypertriglyceridemia on insulin sensitivity and pancreatic beta cell function 28. From a clinical perspective, hypertriglyceridemia may occur as much as 10 years before type 2 diabetes mellitus incidence 29. Hypertriglyceridemia leads to insulin resistance, which in turn causes compensatory hyperinsulinemia, enhancing flux of free fatty acids 30. The increase of flux of free fatty acids then promotes hepatic triglyceride synthesis, aggravating hepatic steatosis, hepatic insulin resistance, and hypertriglyceridemia, resulting in a vicious cycle 31. Moreover, hypertriglyceridemia subsequently causes significant deposition of fat droplets in the islets 32, impairing glucose-induced insulin secretion 33, eventually contributing to the occurrent of diabetes. On the other hand, low levels of TG were also associated with increased risk of incident type 2 diabetes mellitus. The underlying pathogenesis may partially relate to “TG paradox”, a situation with unexpected low TG concentration in the presence of high risk of insulin resistance or type 2 diabetes mellitus in people of African descent 23, 34, 35, which may be explained by the inhibition of insulin sensitive lipase and the reduction in the release of free fatty acids from adipose tissue secondary to hyperinsulinemia 23, 34, 36. Moreover, carriers of PNPLA3 148M allele showed lower TG levels but more insulin resistant and more susceptible to diabetes 22, which may also link with the high incident diabetes with low TG concentration in this study, however, the DNA sequences of the participants were not genotyped in the current study. Actually, there were other evidences indicating that very low levels of TG were detrimental in health and diseases. A cross sectional survey in China reported that low triglyceride increased risk of cardiovascular diseases (CVD) among those patients with 15 and more years of duration of diabetes 37, and another study suggested that low concentration of TG was a predictor of cardiac death in patients with heart failure 38. Besides, Li W et al. and Jain M et al. demonstrated that low serum TG levels worsened the prognosis in acute ischemic stroke 39, 40. Lower TG levels were also linked with higher hemorrhagic stroke risk in a prospective cohort study among 27,937 women 41 and more severe motor performance in Parkinson's disease 42 both of which are closely associated with diabetes 43-45. Moreover, Statins, with the effect of decrease plasma TG concentration 46, 47 and protective against NAFLD 48-51 and the development of liver fibrosis attributed to NAFLD 52, has been reported to increased risk of new-onset diabetes by 12% (OR, 1.12) in an intensive-dose therapy compared with moderate-dose statin therapy 53. As statins reduce plasma TG levels in a dose-dependent manner 46, 47, these findings suggested that inappropriately low levels of TG were linked with a higher occurrence of diabetes, in parallel with an inverse association between baseline TG (< 53mg/dl) and incident type 2 diabetes mellitus in the current study. Accordingly, these findings suggested that TG may be a double-edged sword, both too low and too high levels of TG are adverse in terms of incident type 2 diabetes mellitus in males with NAFLD; additionally, when stains are used in males with NAFLD, the accompanied increased risk of incident type 2 diabetes mellitus and other detrimental effects should be taken into account.

The strengths of this study included the exclusion of individuals with either HbA1c ≥ 5.7% or an impaired FPG level, thus, these findings may preferably apply to glycemic healthy males diagnosed with NAFLD at baseline. However, this study also had several limitations. This study is performed only in the normoglycemic male Japanese population with NAFLD and without other known liver diseases or any medication usage, and the relationship between baseline TG and incident type 2 diabetes mellitus was uncertain in other ethnicities, areas or in females. Therefore, the U-shaped association may be only applicable to this specific population, and the extrapolation of this finding to other populations should be taken with caution. Additionally, the oral glucose tolerance test was not performed and the occurrences of incident type 2 diabetes mellitus (outcome) might have been partially underestimated in the present study.

## Conclusions

To conclude, this study is the first to report a U-shaped relationship between baseline TG levels and the risk of incident type 2 diabetes mellitus in a Japanese normoglycemic male population with NAFLD. Either very high or very low TG levels were detrimental. Nevertheless, this finding may be only applicable to this specific population, and the extrapolation to other populations should be taken with caution.

## Figures and Tables

**Table 1:** Baseline Characteristics of the participants according to TG tertiles.

Baseline Characteristic	T1	T2	T3	P-value
N	404	409	408	
Age (years)	43.44 (8.23)	43.78 (8.27)	43.17 (7.51)	0.559
Body weight (kg)	71.79 (9.99)	74.07 (10.91)	75.78 (10.33)	<0.001
Waist circumference (cm)	84.48 (7.11)	86.41 (7.65)	87.79 (6.96)	<0.001
BMI (kg/m2)	24.62 (2.81)	25.35 (3.13)	25.93 (2.86)	<0.001
ALT (IU/L)	25.00 (20.00-35.00)	28.00 (22.00-41.00)	31.00 (24.00-45.00)	<0.001
AST (IU/L)	20.93 (7.32)	22.38 (8.55)	23.57 (9.08)	<0.001
GGT (IU/L)	20.00 (15.00-26.00)	23.00 (18.00-32.00)	28.00 (21.00-40.00)	<0.001
Ethanol consumption (g/week)	1.00 (0.00-36.00)	2.80 (1.00-54.00)	9.33 (1.00-60.00)	0.017
HDL-cholesterol (mg/dL)	48.87 (9.88)	43.87 (8.18)	39.49 (8.59)	<0.001
Total Cholesterol (mg/dL)	194.25 (29.11)	207.84 (31.26)	219.48 (31.87)	<0.001
TG (mg/dL)	66.12 (16.36)	115.16 (14.59)	207.77 (65.96)	<0.001
HbA1c (%)	5.16 (0.27)	5.15 (0.28)	5.16 (0.28)	0.957
FPG (mg/dl)	93.75 (4.41)	94.01 (4.53)	94.04 (4.38)	0.6
SBP (mmHg)	121.31 (13.56)	123.57 (14.50)	124.21 (13.60)	0.008
DBP (mmHg)	76.56 (9.71)	78.07 (9.89)	78.72 (9.35)	0.005
Regular exercise				0.181
No	334 (82.67%)	338 (82.64%)	354 (86.76%)	
Yes	70 (17.33%)	71 (17.36%)	54 (13.24%)	
Smoking				0.001
never	162 (40.10%)	170 (41.56%)	139 (34.07%)	
past	124 (30.69%)	122 (29.83%)	102 (25.00%)	
current	118 (29.21%)	117 (28.61%)	167 (40.93%)	
Father or mother with diabetes				0.237
No	393 (97.28%)	400 (97.80%)	391 (95.83%)	
Yes	11 (2.72%)	9 (2.20%)	17 (4.17%)	

Continuous variables are presented as mean ± S.D. or as median (Q1-Q3). Categorical data are presented as number (percentage). BMI: Body mass index; TG: Triglycerides; TC: Total Cholesterol; FPG: Fasting plasma glucose; SBP: Systolic blood pressure; DBP: Diastolic blood pressure.

**Table 2:** Univariate associations between baseline factors and incident type 2 diabetes.

Factors at baseline	HR	95%CI	P value
TG (mg/dL)	1.01	(1.00, 1.01)	0.0001
Age (years)	1.06	(1.02, 1.10)	0.0064
Ethanol consumption (g/week)	1	(0.99, 1.00)	0.4188
BMI (kg/m2)	1.16	(1.07, 1.26)	0.0003
Waist circumference (cm)	1.08	(1.04, 1.11)	<0.0001
ALT (IU/L)	1.01	(1.00, 1.03)	0.0711
AST (IU/L)	1.03	(0.99, 1.06)	0.1258
Body weight (kg)	1.04	(1.01, 1.07)	0.0038
GGT (IU/L)	1.01	(1.00, 1.01)	0.1734
HDL-cholesterol (mg/dL)	0.98	(0.95, 1.02)	0.3289
Total Cholesterol (mg/dL)	1	(0.99, 1.01)	0.3916
HbA1c (%)	26.42	(7.02, 99.47)	<0.0001
FPG (mg/dl)	1.06	(0.98, 1.15)	0.1417
SBP (mmHg)	1.02	(0.99, 1.04)	0.1897
DBP (mmHg)	1.02	(0.99, 1.06)	0.172

CI: confidence interval; BMI: Body mass index; FPG: Fasting plasma glucose; SBP: Systolic blood pressure; DBP: Diastolic blood pressure.

**Table 3:** Association between baseline TG and incident diabetes

	Incident diabetes							
	Crude model			Adjusted model I			Adjusted model II	
	HR (95%CI)	P value		HR (95%CI)	P value		HR (95%CI)	P value
TG per 10 mg/dl increment (continuous)	1.066 (1.032, 1.102)	0.00011		1.068 (1.033, 1.104)	0.00013		1.085 (1.039, 1.132)	0.00019
TG tertiles								
T1(≤ 90mg/dl)	1			1			1	
T2(91-141mg/dl)	1.374 (0.523, 3.611)	0.51883		1.23 (0.46, 3.25)	0.68204		1.418 (0.489, 4.109)	0.51978
T3(≥142 mg/dl)	2.867 (1.224, 6.713)	0.01527		2.60 (1.10, 6.16)	0.02991		2.871 (1.001, 8.234)	0.04984

Crude model adjusted for none. Adjusted model I adjusted for Age, BMI (body mass index, kg/m2), Waist circumference (cm); Body Weight (kg). Adjusted model II adjusted for model I plus ethanol consumption (g/week), ALT, AST, regular exercise, GGT, HDL-cholesterol, Total Cholesterol, HbA1c, smoking, Fasting plasma glucose, systolic blood pressure, diastolic blood pressure, and father or mother with diabetes.

**Table 4:** Threshold effect analysis of TG on incident diabetes using piece-wise linear regression.

	Incident diabetes							
	Crude model			Adjusted model I			Adjusted model II	
	HR (95%CI)	P value		HR (95%CI)	P value		HR (95%CI)	P value
Per 10mg/dl increment								
TG<53mg/dl	0.384 (0.227, 0.649)	0.0003		0.321 (0.186, 0.555)	< 0.0001		0.413 (0.220, 0.778)	0.0062
TG>53mg/dl	1.080 (1.046, 1.115)	< 0.0001		1.083 (1.048, 1.118)	< 0.0001		1.091 (1.046, 1.137)	< 0.0001

Crude model adjusted for none. Adjusted model I adjusted for Age, BMI (body mass index, kg/m2), Waist circumference (cm); Body Weight (kg). Adjusted model II adjusted for model I plus ethanol consumption (g/week); ALT, AST, regular exercise, GGT, HDL-cholesterol, Total Cholesterol, HbA1c, smoking, Fasting plasma glucose, systolic blood pressure, diastolic blood pressure, and father or mother with diabetes.
